# Evaluation of the main regulators of systemic iron homeostasis in pyruvate kinase deficiency

**DOI:** 10.1038/s41598-023-31571-2

**Published:** 2023-03-16

**Authors:** Anna Zaninoni, Roberta Marra, Elisa Fermo, Dario Consonni, Immacolata Andolfo, Anna Paola Marcello, Barbara Eleni Rosato, Cristina Vercellati, Wilma Barcellini, Achille Iolascon, Paola Bianchi, Roberta Russo

**Affiliations:** 1 Hematology Unit, Pathophysiology of Anemias Unit, Foundation IRCCS Ca’ Granda Ospedale Maggiore Policlinico, Milan, Italy; 2 Department of Molecular Medicine and Medical Biotechnology, University Federico II, Naples, Italy; 3 CEINGE – Biotecnologie Avanzate Franco Salvatore, Naples, Italy; 4 Epidemiology Unit, Foundation IRCCS Ca’ Granda Ospedale Maggiore Policlinico, Milan, Italy

**Keywords:** Haematological diseases, Metabolism

## Abstract

Iron homeostasis and dyserythropoiesis are poorly investigated in pyruvate kinase deficiency (PKD), the most common glycolytic defect of erythrocytes. Herein, we studied the main regulators of iron balance and erythropoiesis, as soluble transferrin receptor (sTfR), hepcidin, erythroferrone (ERFE), and erythropoietin (EPO), in a cohort of 41 PKD patients, compared with 42 affected by congenital dyserythropoietic anemia type II (CDAII) and 50 with hereditary spherocytosis (HS). PKD patients showed intermediate values of hepcidin and ERFE between CDAII and HS, and clear negative correlations between log-transformed hepcidin and log-EPO (Person’s r correlation coefficient =  − 0.34), log-hepcidin and log-ERFE (r =  − 0.47), and log-hepcidin and sTfR (r =  − 0.44). sTfR was significantly higher in PKD; EPO levels were similar in PKD and CDAII, both higher than in HS. Finally, genotype–phenotype correlation in PKD showed that more severe patients, carrying non-missense/non-missense genotypes, had lower hepcidin and increased ERFE, EPO, and sTFR compared with the others (missense/missense and missense/non-missense), suggesting a higher rate of ineffective erythropoiesis. We herein investigated the main regulators of systemic iron homeostasis in the largest cohort of PKD patients described so far, opening new perspectives on the molecular basis and therapeutic approaches of this disease.

## Introduction

Pyruvate kinase deficiency (PKD), due to bi-allelic mutations in *PKLR* gene (1q21), is the most common enzyme-related glycolytic defect that results in red blood cell (RBC) hemolysis^1^. Pyruvate kinase enzyme plays a key role in glycolysis, converting phosphoenolpyruvate to pyruvate, upon which RBC metabolism completely hinges. Lack of PK enzyme affects RBC ATP production, causing RBC damage, and the consequent trapping of defective cells by splenic and hepatic capillaries. Patients with PKD present with varying degrees of clinical manifestations, ranging from mild or asymptomatic well-compensated hemolysis to severe transfusion-dependent anemia from birth; complications include iron overload, pulmonary hypertension, endocrinopathies, liver failure, biliary disease, and extramedullary hematopoiesis^2^. The high phenotype variability reflects the molecular heterogeneity of the disease, with more than 400 pathogenic variants reported in *PKLR* gene^3^. A more severe phenotype, including rare complications, is usually observed in patients carrying non-missense mutations or missense mutations characterized by severely decreased protein stability^4^.

Intriguingly, PKD patients have been shown to develop iron overload even in absence of transfusions, suggesting dyserythropoietic features^5^. Moreover, recent studies showed that a quote of patients with clinical suspicion of congenital dyserythropoietic anemia (CDA) had a conclusive molecular diagnosis of chronic anemia arising from enzymatic defects, mainly PKD^6^. The presence of bone marrow abnormalities and ineffective erythropoiesis in these patients led to misdiagnosis, further supporting this evidence^7^.

Human PK comprises four isozymes (L, R, M1, M2). R-PK is the only one found in normal mature red cells. Hematopoietic stem cells and progenitor cells express M2-PK, which switches to R-PK in cells of the erythroid lineage, where it is constantly synthesized during erythroid cell maturation^7^. Thus, it was suggested that metabolic abnormalities in R-PK deficiency could alter the differentiation of erythroid progenitors into mature erythrocytes. Indeed, it was reported that hematopoiesis was enhanced in the spleen of PK-deficient patients^8^.

Evaluation of hepcidin, the master regulator of iron balance, has a key role in determining iron status due to ineffective erythropoiesis. It was demonstrated that the pathogenesis of iron overload in iron-loading anemias, such as CDA type II (CDAII) and beta-thalassemia, is related to the over-expression of the erythroblast-derived hormone erythroferrone (ERFE)^9^, leading to hepcidin suppression^10–12^. Dysregulation of the EPO-ERFE-hepcidin pathway has been recently reported in a group of patients with congenital hemolytic anemia including PKD^13^.

Herein, we investigated the pathophysiological basis of iron overload and ineffective erythropoiesis in a larger cohort of PKD patients, stratified by *PKLR* genotype, compared either with the model of a structural RBC membrane defect, such as hereditary spherocytosis (HS), or with the typical model of dyserythropoietic anemia with ineffective erythropoiesis, as CDAII.

## Results

### Analysis of erythropoiesis biomarkers in different inherited anemias

We examined 41 PKD, 42 CDAII, and 50 HS patients who differed for a number of characteristics (Table 1). In PKD patients, median age at the study was 26 years, hemoglobin (Hb) level was 9.1 g/dL, reticulocytes absolute number was 156 × 10^9^/L, and ferritin level was 546 ng/mL. Twenty-nine PKD patients received at least three transfusions during their life, 14 of them were transfusion dependent (≥ 6 transfusions/year), 12 underwent splenectomy, and 15 patients required chelation therapy for iron overload developed also in absence of transfusions. Comparing with the other patient groups, PKD patients were younger, had Hb level similar to CDAII but lower than HS and showed intermediate median reticulocytes number between CDAII and HS patients (Table 1).

**Table 1:** Clinical and hematologic data of patients.

	CDAII (N = 42)	PKD (N = 41)	HS (N = 50)	Reference range	P-value
Center (Milan/Naples)	14/28	24/17	50/0	–	< 0.001
Gender (M/F)	16/26	25/16	32/18	–	0.03
Age (years)	33 (1–73)	26 (1–73)	40 (0–89)	–	0.01
Age < 18 years (N, %)	14 (33)	18 (44)	5 (10)	–	0.001
Hematochemical and biochemical data					
Hb (g/dL)	9.5 (4.7–12.3)	9.1 (5.5–14.5)	13.0 (7.2–17.3)	11.0–16.0	< 0.001
MCV (fL)	89.2 (60.0–110.0)	94.4 (62.8–130.2)	88.3 (72.9–105.0)	77.0–95.0	0.007
Reticulocytes (× 10^9^/L)	62 (7.6–223)	156 (139–1124)	242 (29–571)	20–100	< 0.001
Unconj. bilirubin (mg/dL)	1.6 (0.1–11.5)	2.6 (0.4–18.3)	1.9 (0.5–10.8)	0.2–0.8	0.04
LDH (U/L)	285 (131–718)	361 (140–3236)	220 (144–564)	115–211	0.04
Iron balance					
Serum ferritin (ng/mL)	344 (48–2461)	546 (59–4490)	337 (33–1667)	22–275	0.03
Serum transferrin (mg/dL)	189 (111–290)	209 (78–419)	233 (146–795)	200–360	< 0.001
Transferrin saturation (%)	81.0 (13–113)	59.7 (22–139)	50.7 (23–129)	15–39	0.03
Transfusion regimen, treatment, and surgical intervention					
Never transfused (N, %)	23 (57)	10 (26)	41 (85)	–	< 0.001
Transfusion (N, %)	17 (43)	29 (74)	7 (15)	–	
Transfusion needs				–	0.03
TD (N, %)	5 (29)	14 (48)	0	–	
TD-pre splenectomy (N, %)	2 (12)	4 (14)	0	–	
Occasional (N, %)	10 (59)	9 (32)	7 (100)	–	
Splenectomy (N, %)	15 (36)	12 (29)	7 (14)	–	0.03
Chelation (N, %)	13 (31)	15 (37)	0	–	< 0.001
Chelation at study time (N, %)	9 (21)	13 (32)	0	–	< 0.001

Clinical information and laboratory data are referred at sampling. For quantitative variables data are presented as median (range).

*TD* transfusion dependency.

P-values from chi-squared (categorical variables) or Kruskal–Wallis (quantitative variables).

**Figure 1:**
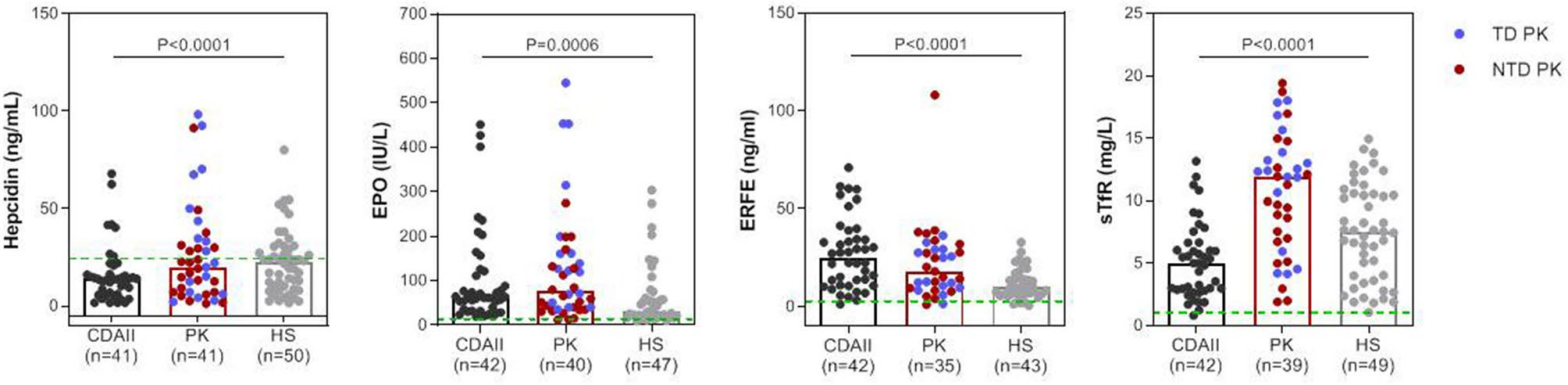
Distribution of hepcidin, EPO, ERFE, and sTfR in CDAII, PKD, and HS patients. Median values and range are reported. Green lines represent median control’s values. Transfusion-dependent (TD—blue filled circle) and not transfusion-dependent (NTD—red filled circle) PKD patients are shown. Kruskal–Wallis test; P-values and reference ranges for each variable are reported in Table 2.

**Figure 2:**
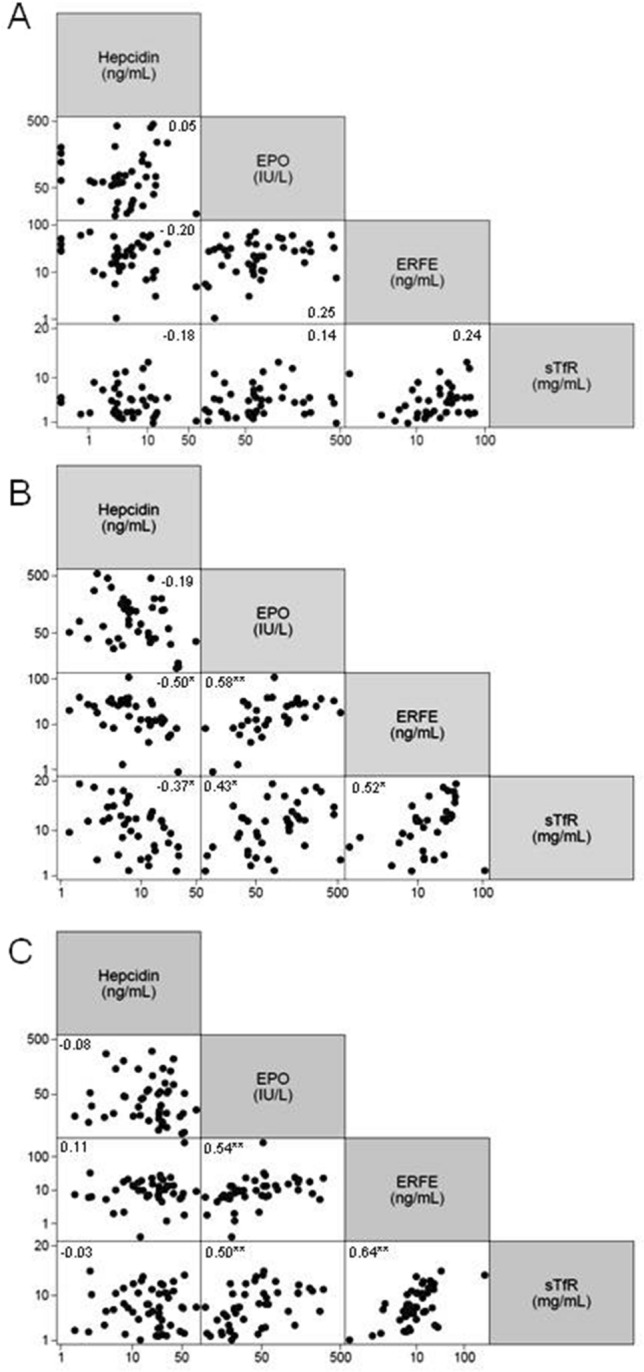
Pairwise correlations between selected variables in CDAII (**A**), PKD (**B**), and HS patients (**C**). Pearson’s r correlation coefficients are indicated in the figure. *P < 0.05 and **P < 0.001. Hepcidin, EPO, and ERFE were ln-transformed.

All patients showed ERFE, EPO, and sTfR levels clearly increased respect to normal controls. On the contrary, hepcidin levels were lower than normal in both PKD and CDAII (Table 2). Interestingly, the comparison among the three groups showed that PKD patients had intermediate values of hepcidin and ERFE between CDAII and HS. No differences were observed regarding EPO levels in PKD and CDAII patients, both higher than in HS. Finally, sTfR was significantly higher in PKD compared with the other hemolytic patients. These results were confirmed in multiple regression analysis adjusted for center (data not shown). The analysis of single data distribution, although showing an expected variability, highlighted that PK deficient patients have intermediate values between CDAII and HS, suggesting that PK deficiency displays ineffective erythropoiesis features as in CDAII, independently from transfusion status (Table 2, Fig. 1). In PKD and HS patients we observed positive correlations between EPO, ERFE and sTfR and a negative correlation between hepcidin and ERFE. A similar pattern, although weaker, was observed in CDAII patients (Fig. 2A–C).

**Table 2:** Iron homeostasis biomarkers evaluation in patients with PKD, CDAII, and HS.

	PKD (N = 41)	CDAII (N = 42)	HS (N = 50)	P^a^	P^b^	P^c^	Controls (N = 25)
Hepcidin (ng/mL)	7.2 (1.3–49.4)	3.7 (0.3–67.9)	22.5 (1.6–80.0)	0.002	< 0.0001	0.0002	17.2 (3.0–36.0)
ERFE (ng/mL)	18.0 (0.9–108.0)	24.8 (1.1–70.9)	9.9 (0.4–266.8)	0.15	< 0.0001	0.005	1.0 (0.3–7.6)
EPO (IU/L)	75.6 (12.1–545.0)	62.7 (19.0–451.0)	31.6 (9.7–303.7)	0.44	0.0010	0.001	6.5 (1.9–21.0)
sTfR (mg/L)	11.9 (1.9–19.4)	5.0 (0.8–13.2)	7.5 (1.1–14.9)	< 0.0001	0.004	0.002	0.7 (0.2–1.5)

Values are expressed as median (range).

P^a^—PKD vs CDAII patients; P^b^—CDAII vs HS patients; P^c^—PKD vs HS patients. Mann–Whitney test.

### Iron homeostasis biomarkers and *PKLR* genotype

**Figure 3:**
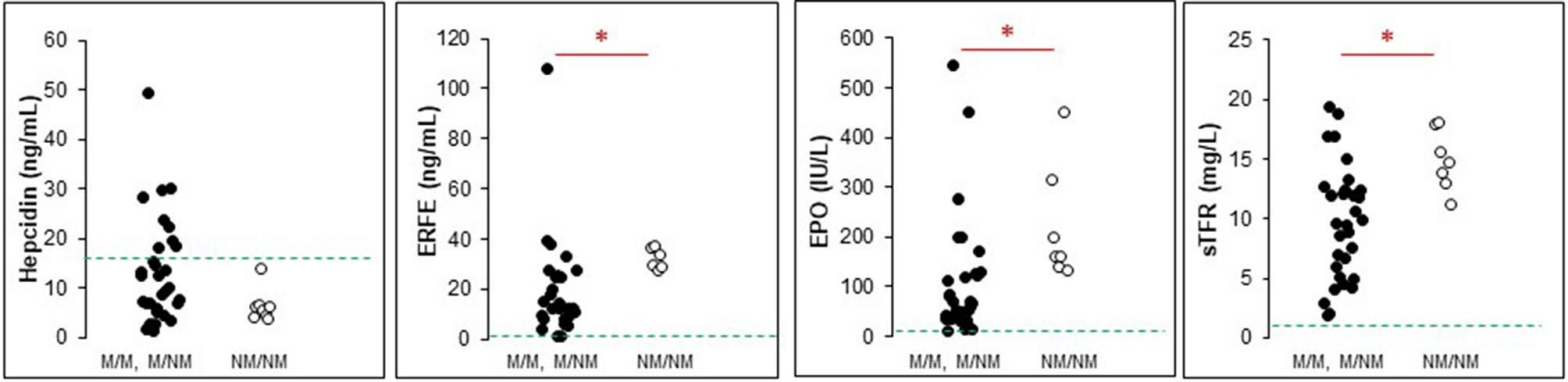
Distribution of hepcidin, EPO, ERFE, and sTfR in PKD patients grouped according with their genotype. Green lines represent median control’s values. *P < 0.001, Mann–Whitney test. Reference ranges for each variable are reported in Table 2.

PKD patients were grouped according with their genotype on the basis of the different types of mutations: missense (M)/M, missense/non-missense (NM) or NM/NM. Twenty-four patients showed M/M, 8M/NM and 8 NM/NM genotype. In one case, PKD genotype was not available, and the patient was excluded by the analysis. NM/NM patients showed lower hepcidin level although not statistically significant (P = 0.07), increased ERFE (P = 0.004), EPO (P = 0.003) and sTfR (P = 0.007) than the other PKD patients suggesting a higher level of ineffective erythropoiesis in this group of patients (Table 3, Fig. 3). Comparable results were observed after considering in the NM/NM group the four patients carrying homozygous c.1529A > G, p.Arg510Gln variant, known to be characterized by severely decreased protein stability^14^.

**Table 3:** Iron homeostasis biomarkers in PKD patients grouped according to PKLR genotype.

	*PKLR* genotype		
	NM/NM (N = 8)	M/M + M/NM (N = 32)	P
Hb (g/dL)	7.8 (7.5–9.9)	9.4 (5.5–14.5)	0.02
Hepcidin (ng/mL)	6.4 (3.9–14.1)	13.0 (1.3–49.4)	0.07
ERFE (ng/mL)	32.3 (27.7–37.4)	19.1 (0.9–108.0)	0.004
EPO (IU/L)	223 (132.2–453.0)	106.2 (12.1–545.0)	0.003
sTfR (mg/L)	14.9 (11.3–18.0)	9.7 (1.9–19.4)	0.007

Values are expressed as median (range).

*NM* non-missense variant, *M* missense variant. Mann–Whitney test.

## Discussion

Pyruvate kinase deficiency is the most common enzyme-related glycolytic defect that results in red cell hemolysis. This disorder is characterized by clinical heterogeneity, which results in a variable degree of hemolytic, non-spherocytic anemia. Manifestations occur from the neonatal period through adult life, causing countless complications^5,15^. The variety of unspecific and overlapping phenotypes with other congenital anemias, particularly between PKD and HS in the mildest forms, and hypo-regenerative anemias such as CDAII, often hampers a correct clinical management of the patients^16^. Presence of inadequate reticulocytosis, erythroblasts in peripheral blood, or of immature forms at bone marrow examination, are not so rare in patients with severe PKD^5^.

Several studies suggested that metabolic abnormalities in PKD could alter the differentiation of erythroid progenitors. Of note, studies on PK-1slc deficient mice showed an increased number of BFU-E, indicating enhanced erythropoiesis. In addition, the presence of apoptotic cells of erythroid lineage in the splenic red pulp suggested that the metabolic disturbance in PKD alters not only the survival of erythrocytes but also the maturation of erythroid progenitors, resulting in ineffective erythropoiesis^17^.

To demonstrate the occurrence of this condition in PKD, we measured the levels of different markers in our cohort of patients, and we compared the results with two different models of congenital hemolytic anemia, CDAII with overt dyserythropoiesis, and HS, characterized by normal erythroid production but increased red cell fragility.

Although most of the analyzed parameters were altered in all the hemolytic patients studied including HS, as expected we found higher level of ineffective erythropoiesis in PKD and CDAII. Indeed, significantly increased levels of EPO were found in PKD patients compared to reference values of healthy subjects. Of note, even with a slight difference, EPO levels were also significantly increased when compared to those of CDAII patients, probably due to a greater hemolytic component and the higher degree of anemia in PKD than CDAII.

It is well known that the concentration of sTfR reflects erythropoiesis rate^18^. Expansion of erythroid precursors to compensate a loss of RBCs leads to an augmented level of sTfR1 due to an increase of highly expressing TfR1^+^ cells^19^. We observed increased levels of sTfR in PKD patients compared not only to CDAII, but also to HS patients. This finding may be partially justified either by the higher ferritin levels found in the PKD subjects respect to the other patients analyzed in this series or by the increased reticulocyte number in both HS and PKD, particularly high after splenectomy in the latter. Of note, sTfR levels in CDAII patients were similar to those previously reported for these patients^12,20^. The increased sTfR levels in HS (in some cases also higher than CDAII patients) are in line with previous reports on different series of HS: a direct correlation between clinical severity and sTfR levels was in fact reported in a large case series of 82 HS^21^. Moreover, increased EPO and sTfR was reported in another case series of 32 HS, also with compensated hemolysis, suggesting that inappropriately high EPO and sTfR may represent a biological characteristic of the disease^22^.

It was already demonstrated that ERFE expression, which is produced by erythroblasts in response to EPO, is increased in conditions characterized by ineffective erythropoiesis, such as CDAII and beta-thalassemia^12,13^, resulting in hepcidin suppression^23^. All our PKD patients showed increased levels of ERFE compared to controls, and slightly decreased values compared to those of CDAII patients. Interestingly, they showed decreased hepcidin values, although to a lesser extent to those observed in CDAII cases, corroborating the observation that ERFE is not the only erythroid regulator that contributes to hepcidin inhibition^23^. Our results agree with a recent study investigating the EPO-ERFE-hepcidin pathway in PKD, HS, and beta-thalassemia patients^13^. In addition, given the extreme phenotypic variability in PKD^24^, we also considered *PKLR* genotype since nonsense *PKLR* variants are known to be associated with severe phenotype^4^, and because of genotype is an independent factor from differences in management approaches, age, or period of the study. Although the limited number of cases due to the rarity of this disease, and the low proportion of NM/NM cases (8/40, 20% of PKD cases), we observed higher level of ineffective erythropoiesis in patients with disruptive variants.

Although the parameter herein analyzed are well-known and widely used markers of ineffective erythropoiesis, they represent only a limited part of the many different direct and indirect players involved in this process^25,26^. Moreover, also iron deposits (as measured with LIC MRI) may put in light dyserythropoietic bases of some disorders^27,28^.

The clinical heterogeneity of the investigated patients in terms of clinical severity, different management approaches, different age at diagnosis, together with selection of the analyzed parameters might represent a bias that accounts for the variability of the results. However, unraveling mechanisms of iron loading and ineffective erythropoiesis in PKD and related disorders by identifying easy-to-access laboratory markers may be useful in differential diagnosis and for new therapeutic perspectives^29^.

## Materials and methods

### Patients, plasma collection, and ELISA assay

Overall, 133 patients with different types of hereditary hemolytic anemias were retrospectively included in the study after obtaining informed consent. For patients under the age of 18, written informed consent was obtained from the parents. The procedures followed were in accordance with relevant guidelines and regulations and with the Helsinki international ethical standards on human experimentation. Clinical diagnosis was based on history, clinical findings, laboratory data, morphological analysis of peripheral blood, and genetic testing^30,31^. Local institution ethical committees approved both the plasma sampling and the collection of patients’ data from Medical Genetics Ambulatory in Naples (University Federico II, DAIMedLab) and Fondazione IRCCS Ca’ Granda Ospedale Maggiore Policlinico of Milan (Milano, Area 2). Plasma samples were collected during routine laboratory investigation from peripheral blood of patients and healthy controls.

Plasma levels of human ERFE (Intrinsic Erythroferrone IE; Intrinsic Lifesciences, La Jolla, California, USA), erythropoietin (EPO) (Quantikine IVD ELISA Human Erythropoietin), and soluble transferrin receptor (sTfR) (Quantikine IVD ELISA Human sTfR, R&D System Minneapolis, Minnesota, USA) were quantified using ELISA kits. Hepcidin evaluation was performed with Intrinsic HEPCIDIN IDxTM (Intrinsic Lifesciences, CA, USA) and Hepcidin-25 ELISA (DRG Instruments GmbH, Germany). In 20/42 CDA patients iron status parameters were previously reported^32^. Prior to the analysis of plasma samples from patients, the reference intervals of both hepcidin assays were evaluated on reference samples for the harmonization of the results obtained by the two different kits used in Naples and Milan respectively. The concentration of each parameter in each sample was determined through the fitting of a four-parameter logistic curve, according to the manufacturer protocols.

### Clinical severity and *PKLR* genotype

For the analysis of the correlation of iron homeostasis markers and clinical severity in PKD, we stratified patients according to transfusions, splenectomy, or iron chelation need. Transfusion needs were classified into the following categories: (a) regularly transfused ≥ 6/year during life; (b) not regularly transfused or not transfused. Intermittent chelation therapy was considered in case of transfusion dependency and/or ferritin levels > 500 ng/mL.

Based on previous observations in literature of the existence of genotype–phenotype correlation in PKD^4^, patients were categorized according to their genotype as follows: (a) missense/missense genotype (M/M); (b) missense/non-missense genotype (M/NM); or (c) non-missense/non-missense genotype (NM/NM). Non-missense pathogenic variants included stop gain, frameshift, in-frame small ins/del/indels, large deletions, and splicing variants; missense mutations also affecting splicing were categorized as non-missense (Supplemental Table 1). For statistical analysis, M/M and M/MN patients were grouped together, given similar clinical phenotype.

### Statistical analysis

Demographic and hematologic data has been expressed as median and range and compared across the three diseases (CDAII, PK deficiency, and HS) by calculating chi-squared and Kruskal–Wallis tests (for categorical or quantitative variables, respectively).

Comparison of quantitative variables across the three diseases were analyzed with Mann–Whitney and Kruskal–Wallis test. Additional multiple regression analyses were performed after adjusting for center. We did not adjust for other variables because they are not confounders (by definition they do not affect inherited disorders)^33^. We calculated Pearson’s r correlation coefficients between selected hematological variables. In regression and correlation analyses hepcidin, EPO and ERFE were ln-transformed. Statistical analyses were performed with Stata 17 (StataCorp. 2021).

## Supplementary Information

Supplementary Table 1.

## Data Availability

All data generated or analysed during this study are included in this published article (and its Supplementary Information files).
