# Supplementary material for: Evaluation of the main regulators of systemic iron homeostasis in pyruvate kinase deficiency
Source: Sci Rep. 2023 Mar 16;13:4395. doi: 10.1038/s41598-023-31571-2 (PMC10020532; doi:10.1038/s41598-023-31571-2)
Supplement: Supplementary file 1 — Supplementary Table 1. [file 41598_2023_31571_MOESM1_ESM.docx]

**Supplementary Table 1. *PKLR* genotype in the PKD patients**

| **Patient code** | **HGVS cDNA-level nomenclature**  allele 1/allele 2 | **HGVS protein-level**  allele 1/allele 2 | **Variant type** |
| --- | --- | --- | --- |
| PKM1 | c.1456C>T/c.1456C>T | p.Arg486Trp/p.Arg486Trp | M/M |
| PKM3 | c.1456C>T/c.1552C>A | p.Arg486Trp/p.Arg518Ser | M/M |
| PKM8 | c.721G>T/c.721G>T | p.Glu241*/p.Glu241* | NM/NM |
| PKM12 | c.1456C>T/c.762G>T | p.Arg486Trp/p.Leu254Phe | M/M |
| PKM13 | c.721G>T/c.284-2A>C | p.Glu241*/- | NM/NM |
| PKM18 | c.1456C>T/c.1515_1518dup | p.Arg486Trp/p.His507Glyfs*14 | M/NM |
| PKM21 | c.1513C>G/ c.965+1G>A | p.Gln505Glu/- | M/NM |
| PKM22 | c.1513C>G/c.965+1G>A | p.Gln505Glu/- | M/NM |
| PKM26 | c.991G>A/c.1015G>T | p.Asp331Asn/p.Asp339Tyr | M/M |
| PKM30 | c.1529G>A/c.1529G>A | p.Arg510Gln/p.Arg510Gln | M/M |
| PKM32 | c.1529G>A/c.1483G>A | p.Arg510Gln/p.Ala495Thr | M/M |
| PKM34 | c.1456C>T/c.721G>T | p.Arg486Trp/p.Glu241* | M/NM |
| PKM36 | c.1456C>T/c.245C>A | p.Arg486Trp/p.Pro82His | M/M |
| PKM37 | c.1456C>T/c.1675C>T | p.Arg486Trp/p.Arg559* | M/NM |
| PKM38 | c.1456C>T/c.1232G>C | p.Arg486Trp/p.Gly411Ala | M/M |
| PKM60 | c.1456C>T/c.1552C>A | p.Arg486Trp/p.Arg518Ser | M/M |
| PKM61 | c.1456C>T/c.1675C>T | p.Arg486Trp/p.Arg559* | M/NM |
| PKM62 | c.1456C>T/c.994G>A | p.Arg486Trp/p.Gly332Ser | M/M |
| PKM79 | c.1529G>A/c.1529G>A | p.Arg510Gln/p.Arg510Gln | M/M |
| PKM80 | c.1456C>T/c.1160A>G | p.Arg486Trp/p.Glu387Gly | M/M |
| PKM86 | c.1456C>T/c.1022G>A | p.Arg486Trp/p.Gly341Asp | M/M |
| PKM87 | c.1456C>T/c.1232G>C | p.Arg486Trp/p.Gly411Ala | M/M |
| PKM88 | c.1529G>A/c.1529G>A | p.Arg510Gln/p.Arg510Gln | M/M |
| PKM89 | c.1529G>A/c.1529G>A | p.Arg510Gln/p.Arg510Gln | M/M |
| PKN22 | c.353A>G/c.353A>G | p.Asn118Ser/p.Asn118Ser | M/M |
| PKN23 | c.1489_1496dup/c.1489_1496dup | p.Ala500Profs*33/p.Ala500Profs*33 | NM/NM |
| PKN24 | c.898G>C/c.898G>C | p.Ala300Pro/p.Ala300Pro | M/M |
| PKN25 | c.1528C>T/c.1528C>T | p.Arg510*/p.Arg510* | NM/NM |
| PKN26 | c.1456C>T/c.1456C>T | p.Arg486Trp/p.Arg486Trp | M/M |
| PKN27 | c.287C>A/c.68_69del | p.Pro96Gln/p.Leu23Cysfs*56 | M/NM |
| PKN28 | c.880G>A/c.880G>A | p.Val294Met/p.Val294Met | M/M |
| PKN29 | c.1675C>T/c.1675C>T | p.Arg559*/p.Arg559* | NM/NM |
| PKN30 | c.1456C>T/c.1456C>T | p.Arg486Trp/p.Arg486Trp | M/M |
| PKN31 | c.469A>G/c.469A>G | p.Thr157Ala/p.Thr157Ala | M/M |
| PKN32 | c.469A>G/c.469A>G | p.Thr157Ala/p.Thr157Ala | M/M |
| PKN33 | n.a/n.a | n.a/n.a | n.a/n.a |
| PKN34 | c.1117-1G>C/c.1117-1G>C | -/- | NM/NM |
| PKN35 | c.823G>A/c.823G>A/ | p.Gly275Arg/p.Gly275Arg | M/M |
| PKN36 | c.1116+2T>G/c.1116+2T>G | -/- | NM/NM |
| PKN37 | c.846del/c.885del | p.Ile282Metfs*39/p.Ala296Profs*25 | NM/NM |
| PKN38 | c.994G>A/c.507+1G>T | p.Gly332Ser/- | M/NM |

All variants are classified as pathogenic, most of them reported in previous studies.

*PKLR* reference transcript: NM_000298.5.

PKM, patients diagnosed at Fondazione IRCCS Ca’ Granda Policlinico Milan; PKN, patients diagnosed at University Federico II, Naples; HGVS, Human Genome Variation Society; M, missense variant; NM, non-missense variant. n.a., not available; - = abnormal splicing.
